# Evaluation of a novel eyelid-warming device in meibomian gland dysfunction unresponsive to traditional warm compress treatment: an in vivo confocal study

**DOI:** 10.1007/s10792-014-9947-3

**Published:** 2014-04-22

**Authors:** Edoardo Villani, Elena Garoli, Veronica Canton, Francesco Pichi, Paolo Nucci, Roberto Ratiglia

**Affiliations:** 1 Department of Clinical Sciences and Community Health, University of Milan, Milan, Italy; 2 University Eye Clinic San Giuseppe Hospital, via San Vittore 12, 20123 Milan, Italy; 3 Ophthalmological Unit, IRCCS Cà Granda Foundation-Ospedale Maggiore Policlinico, Milan, Italy

**Keywords:** Meibomian gland, MGD, Confocal microscopy, Blephasteam, Dry eye, Ocular surface

## Abstract

The purpose of the study was to evaluate the efficacy and safety of wet chamber warming goggles (Blephasteam^®^) in patients with meibomian gland dysfunction (MGD) unresponsive to warm compress treatment. We consecutively enrolled 50 adult patients with low-delivery, non-cicatricial, MGD, and we instructed them to apply warm compresses twice a day for 10 min for 3 weeks and to use Blephasteam^®^ (Laboratoires Thea, Clermont-Ferrand, France) twice a day for 10 min for the following 3 weeks. We considered “not-responders” to warm compress treatment the patients who showed no clinically significant Ocular Surface Disease Index (OSDI) improvement after the first 3 weeks. Clinical and in vivo confocal outcome measures were assessed in the worst eye (lower BUT) at baseline, after 3 weeks, and after 6 weeks. Eighteen/50 patients were not-responders to warm compress treatment. These patients, after 3 weeks of treatment with Blephasteam^®^, showed significant improvement of OSDI score (36.4 ± 15.8 vs 20.2 ± 12.4; *P* < 0.05, paired samples *t* test), increased BUT (3.4 ± 1.6 vs 7.6 ± 2.7; *P* < 0.05), and decreased acinar diameter and area (98.4 ± 18.6 vs 64.5 ± 14.4 and 8,037 ± 1,411 vs 5,532 ± 1,172, respectively; *P* < 0.05). Neither warm compresses nor Blephasteam^®^ determined adverse responses. In conclusion, eyelid warming is the mainstay of the clinical treatment of MGD and its poor results may be often due to lack of compliance and standardization. Blephasteam^®^ wet chamber warming goggles are a promising alternative to classical warm compress treatment, potentially able to improve the effectiveness of the “warming approach.”

## Introduction

Meibomian gland dysfunction (MGD) is a common chronic condition, affecting the tear film and the ocular surface and causing symptoms of eye irritation [1, 2].

Meibomian gland obstruction, due to either terminal duct obstruction or altered secretion, is the most common form of MGD [2]. Eyelid warming, usually achieved with simple warm compresses, is regarded as the mainstay of the clinical treatment of this condition, but its efficacy is affected by lack of standardization, in terms of duration and maintenance of temperature, and by a scarce compliance [3]. In the last few years, different devices have been developed in order to try to improve the heat therapy efficacy [3–8].

In vivo laser scanning confocal microscopy (LSCM) is an emerging technology to study the ocular surface in several conditions, including dry eye and MGD [9–11]. At present, LSCM is showing promising clinical applications [9] and recent studies reported its helpfulness in detecting ocular surface response to treatment [12–14].

The aim of this research is to evaluate the safety and efficacy of Blephasteam^®^ (Laboratoires Thea, Clermont-Ferrand, France) eyelid-warming device in the management of MGD unresponsive to warm compress treatment and to study treatment-related clinical and confocal changes.

## Methods

We consecutively studied 50 adult patients with mild to moderate low-delivery, non-cicatricial, MGD. Written informed consent was obtained from all subjects before enrollment, and this study adhered to the tenets of the declaration of Helsinki. MGD classification and grading were performed according to the 2011 International Workshop on MGD [2, 15]. Briefly, we included patients with symptoms of ocular discomfort (Ocular Surface Disease Index—OSDI—score >12) [16], tear fluorescein break-up time (BUT) <5, mild to moderate meibum quality abnormality (score 11–20, according to Bron’ Scale) [17, 18], and mild to moderate expressibility reduction [15]. Exclusion criteria included blepharitis, ocular allergies, contact lens wear, hyposecretive dry eye, history of ocular trauma or surgery, cicatricial ocular surface diseases, and systemic or topical therapies (tear substitutes excepted) that would interfere with tear film and ocular surface.

All these patients were instructed to perform warm compress treatment twice a day for 10 min.

After 3 weeks of treatment, we defined as “not-responders to warm compress treatment” patients who did not show clinically significant OSDI improvement, based on the previously validated OSDI minimal clinically important difference [19].

Both “responder” and “not-responder” patients were then instructed to use Blephasteam^®^ (Laboratoires Thea, Clermont-Ferrand, France) twice a day for 10 min, following the manufacturer instructions, for the following 3 weeks.

All the visits (screening and enrollment—V0, visit at day 21 ± 2—V1, and visit at day 42 ± 2—V2) included the same procedures, performed in the order suggested by the 2007 International Dry Eye Workshop [20]: OSDI questionnaire, fluorescein BUT, fluorescein corneal staining (quantified using the CLEK scheme) [21], Schirmer test without topical anesthesia, meibomian gland expression, and LSCM (HRT II Corneal Rostock Module, Heidelberg Engineering GmbH, Dossenheim, Germany) of meibomian glands. Confocal examination was performed at the lower eyelid margin, following a previously published procedure [22–24]. Meibomian acinar units were analyzed, quantifying their density, mean diameter, and area.

No changes in the concomitant medications, including artificial tears, were allowed during the study period.

The outcome measures were assessed in the worst eye, defined as the eye with the lower BUT.

### Statistical analysis

The statistical analysis was conducted with commercial software (SPSS for Windows, ver. 12.0; SPSS, Chicago, IL, USA). The comparisons between consecutive visits were performed using the *t* test for repeated measures for parametric variables and with the Wilcoxon test for non-parametric variables. The comparisons between “responders” and “not-responders” to warm compress treatment were done using the *t* test for independent samples for parametric variables and the Mann–Whitney *U* test for non-parametric variables. The minimum criterion for tests of significance was *P* < 0.01.

## Results

The 50 enrolled patients (31 females and 19 males) had a mean age of 64 ± 12 years.

After 3 weeks of warm compress treatment, 18 patients (36 %) were classified as “not-responders” and 32 (64 %) as “responders.”

No significant differences were found between the baseline characteristics of “responders” and “not-responders.”

Neither warm compresses nor Blephasteam^®^ caused adverse events or problems of tolerability in our patients.

**Fig. 1:**
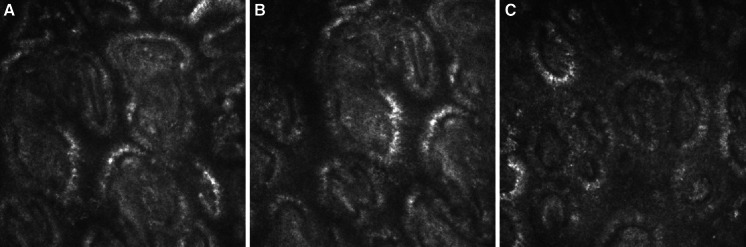
LSCM images of meibomian glands’ acinar units in a patient not-responder to warm compress treatment: baseline (**a**), V1—after 3 weeks of warm compresses (**b**), and V2—after 3 weeks of treatment with Blephasteam^®^(**c**)

In the “not-responders” group, comparing V0 to V1, we found no significant treatment-related (warm compresses) improvement in clinical findings. Furthermore, in this same group, we observed significant improvement of OSDI score (Table 1), increase of BUT (Table 2), and decrease of both acinar mean diameter (Table 3) and area (Table 4) (Fig. 1) from V1 to V2.

**Table 1:** OSDI score during follow-up, patients responders to and not-responders to warm compress treatment

	V0	V1	V2	*P (V0 vs V1)	*P (V1 vs V2)
Responders (*n* = 32)	36.3 ± 17.1	22.7 ± 13.1	20.5 ± 14.2	<0.05	n.s.
Not-responders (*n* = 18)	38.2 ± 15.5	36.4 ± 15.8	20.2 ± 12.4	n.s.	<0.05
**P	n.s.	<0.05	n.s.		

*n.s.* Not significant

* *P* by *t* test for repeated measures

** *P* by *t* test for independent samples

**Table 2:** BUT (seconds) during follow-up, patients responders to and not-responders to warm compress treatment

	V0	V1	V2	*P (V0 vs V1)	*P (V1 vs V2)
Responders (*n* = 32)	3.3 ± 1.8	6.7 ± 2.1	7.2 ± 2.2	<0.05	n.s.
Not-responders (*n* = 18)	3.8 ± 1.4	3.4 ± 1.6	7.6 ± 2.7	n.s.	<0.05
**P	n.s.	<0.05	n.s.		

*n.s.* Not significant

* *P* by *t* test for repeated measures

** *P* by *t* test for independent samples

**Table 3:** LSCM assessment of mean acinar diameter (µm) during follow-up, patients responders to and not-responders to warm compress treatment

	V0	V1	V2	*P (V0 vs V1)	*P (V1 vs V2)
Responders (*n* = 32)	108.3 ± 19.4	84.2 ± 17.6	77.5 ± 18.0	<0.05	n.s.
Not-responders (*n* = 18)	104.8 ± 15.1	98.4 ± 18.6	64.5 ± 14.4	n.s.	<0.05
**P	n.s.	<0.05	n.s.		

*n.s.* Not significant

* *P* by *t* test for repeated measures

** *P* by *t* test for independent samples

**Table 4:** LSCM assessment of mean acinar area (µm^2^) during follow-up, patients responders to and not-responders to warm compress treatment

	V0	V1	V2	*P (V0 vs V1)	*P (V1 vs V2)
Responders (*n* = 32)	8,645 ± 1,980	6,026 ± 1,883	5,879 ± 1,820	<0.05	n.s.
Not-responders (*n* = 18)	8,276 ± 1,691	8,037 ± 1,411	5,532 ± 1,172	n.s.	<0.05
**P	n.s.	<0.05	n.s.		

*n.s.* Not significant

* *P* by *t* test for repeated measures

** *P* by *t* test for independent samples

Fluorescein staining, meibomian gland expressibility, and meibum quality showed no significant differences during the follow-up (Wilcoxon test).

## Discussion

MGD, specifically in the low-delivery, non-cicatricial form, is an increasingly prevalent affliction/with potentially severe detriments to well-being [1]. Dedicated and reliable methods to monitor and effective approaches to manage the disease are still partially unmet needs. LSCM offers new opportunities to perform in vivo, non-invasive examinations of meibomian glands. This technology has proven to have the potential to diagnose MGD with high sensitivity and specificity [25] and to explore the different patterns of the disease, providing new information on the pathogenetic process and the acinar morphological changes [22–24]. Moreover, the previously hypothesized [12] suitability of LSCM to detect and quantify the MGD response to treatment may be confirmed by the present study. Our results showed agreement between clinical (symptoms and BUT) and confocal changes, in the absence of significant variations of traditional meibomian expression scores. These interesting data suggest the need for future studies to compare confocal and clinical examination of meibomian glands and to confirm the utility of incorporating LSCM analysis in the assessment of patient response to therapy.

Our study confirms the usefulness of the well-known and broadly accepted [3] eyelid-warming approach to MGD, but it also highlights that this treatment may be ineffective in 1/3 of the patients, although carefully selected. The good clinical and morphological response to Blephasteam^®^ of subjects “not-responder” to warm compress treatment suggests that ineffectiveness may be due to poor standardization and compliance more than to poor rationale. Medical devices dedicated to eyelid warming try to bridge this gap in the management of MGD patients. Blephasteam^®^ is an electrical pair of goggles that provides warmth and steam, with controlled treatment temperature and duration. Previous studies in healthy volunteers [7, 8] already showed that this device, compared to traditional warm compresses, provides longer warming of the eyelid margin without any adverse ocular response. Our results show its safety and efficacy in MGD patients and its potential superiority to warm compress treatment in ideal candidates to warming therapy.

In conclusion, new technologies as LSCM and eyelid-warming devices promise to play an important role in the management of MGD, improving the effectiveness of non-pharmacological treatment of this common condition.
